# Dr. Hamilton's Observations on the Use and Abuse of Mercurial Medicines in Various Diseases

**Published:** 1820-06-01

**Authors:** 


*Observations on the Use and Abuse of Mercurial Medicines in various Diseases.* By JOHN HAMILTON, M.D. jun. Fellow of the Royal College of Physicians, and Professor of Midwifery in the University of Edinburgh. One Vol. 8vo. pp. 222. Edinburgh, 1819.

——— Spargere voces
In vulgum ambiguas.

As the laws of Medical Science are by no means so fixed and certain as those of Physics, so the exceptions to general rules are much more numerous in the former than in the latter. The natural, unavoidable, and perhaps salutary consequence of this is, that a wider field for speculation and disputation is opened in medicine than in any other branch of human knowledge. Among the phenomena resulting also from this state of things, we every now and then observe an individual, whether impelled by vanity, or under the influence of a certain obliquity of intellect, come before the public with a port-folio of the above mentioned *exceptions,* industriously collected from every point of the literary horizon, with the avowed purpose of beating down all the general rules which the experience of mankind had constructed, and setting up these, his mis-shapen dogmas, to reign in their place!

It is evident, that the more universal the law which is thus to be demolished, the greater the honour in attacking it—these champions, acting on the maxim, perhaps, that " in great attempts 'tis glorious ev'n to fail;" or, more probably, adopting the motto of the most ancient and renowned of all *radical* reformers—

" Better to reign in Hell than serve in Heaven."
*Paradise Lost.*

Although a very early oblivion awaits, in general, upon these *exceptionable* doctrines, yet it is necessary to circulate the antidote with the poison, in order that as much good as possible may be elicited by the collision. With this view, we shall take the present opportunity of stemming the torrent of " abuse of mercurial medicines," which has lately issued from the professorial chair of our learned author, with a freedom which must convince him that *we,* at least, are not to be misled or intimidated by those *ex cathedra* denunciations.

When a teacher leaves his chair, and enters the territories of that rigid republic, the PRESS, he must expect to find his doctorial passports to implicit credence, of no avail. He addresses his equals, who will scrutinize his facts, and analyse his theories, without the least respect of persons; and if they are found wanting in the balance, they will be set aside with as little ceremony as if they were the products of the humblest member of the profession.

About eight years ago, Dr. Mills, of Dublin, published a work on the same subject which the present volume embraces; representing mercury as the box of Pandora, whence all kinds of disorders were let loose upon society. This would have been a rich treat for Dr. Hamilton; but (ominous for the latter!) the book dropped still-born from the press, and scarcely does a catalogue "tell where it lies." Now Dr. Mills has published several works since that period, all of which were instantly read by the profession, and continue to be quoted and referred to, as meritorious publications. What are we to infer from this? Most indubitably, that the "abuse of mercurial medicines" has no effect on the public mind, and that the remedy maintains its honestly acquired reputation, in spite of all the scandal and defamation which can be showered upon it.

That a medicine which, in one shape or other, is of such universal application as mercury, should be misused by ignorance, carelessness, or temerity, cannot be wondered at; but that these *abuses* should be blazoned forth as the common and general effects of the remedy, by clamorous terrorists, is somewhat derogatory to that cool, and chaste, and inductive philosophy, by which the present race is anxious to be distinguished.

We shall open our critical analysis of Dr. Hamilton's work with the following curious passage.

> "In *feverish disorders* there is neither any apparent change upon the sensible qualities of the blood, nor any unusual flow of the ordinary secretions; while in *inflammatory affections* the sensible qualities of the blood are materially altered, and either some of the *secretions* are furnished in *greater abundance*, or topical congestions, that is, obstructions in the vessels of particular parts, take place." 6

Passing over the obscurity of Dr. H's distinction between "feverish disorders" and "inflammatory affections," we would ask him on what authority he states that the sensible qualities of the blood are unaffected in the *former*, and materially altered in the *latter*? He can give, we apprehend, no authority for this assertion; and the inference is, that he knows nothing about the matter.*

* See our review of Thackrah on the blood, No. vi.

We would also ask Dr. H. in which of the phlegmasiæ is there any increase of the natural secretions? In the same page he asserts, that the mercurial action on the system "is attended with the most obvious of the circumstances which arise from inflammation." Thus, *topical congestions* result from the mercurial influence on the system! But in the very next passage he gives almost a flat contradiction to his own statement.

"In inflammatory complaints, topical congestions occur more frequently than increase of secretions [yes, about ten thousand times for once] while the *reverse of this* happens where the inordinate action of the heart and arteries is occasioned by the use of mercury." P. 7.

Such is the medical logic by which Professor Hamilton labours to identify mercurial action with inflammation — nay, to prove that it is worse than inflammation — namely, by a confession that the phenomena attendant on the one, are generally the reverse of those on the other!

"There is another remarkable difference (says the Professor); in inflammatory diseases, the *muscular strength*, in many instances, continues *unimpaired* till towards the termination of the complaint. Thus every practitioner knows, that individuals labouring under pleurisy have walked several miles within a few hours of death. But from the time that the influence of mercury becomes evident, *the general strength declines rapidly*." P. 7.

As our reasoning powers can only analyse, compare, or combine the evidences of our senses, so, if these last fail to transmit faithful images of things to the sensorium, our judgements must be defective. We would not hold in great estimation a treatise on music from a man born deaf; nor would we go to a man affected with congenital blindness to ask his opinion on the correctness of a portrait. In the science of medicine, if the phenomena of disease, from some cause or other, should happen to be inverted during their transmission to the seat of reason in any individual; if the symptoms of strength, for instance, presented themselves in the form of debility, and those of weakness in the garb of vigour, we might very justly expect that the train of false perceptions would be followed by a train of erroneous conclusions.

In the first place, we appeal to "every practitioner," whether he has ever seen a man within a few hours of death from pulmonic inflammation, walk several miles? If, in a disastrous military retreat, or, in fact, under any pressing emergency, a sick man were forced to walk several miles, and in a few hours afterwards died, with evidences of thoracic inflammation, on dissection, would any man in his senses conclude from this, that the sick man's strength "continued unimpaired" the while; and that his death was no way accelerated by this muscular exertion? No. "Every practitioner" is well aware that when thoracic inflammation has brought its victim to within a few hours of his final goal, the muscular powers are not only greatly prostrated, but every exertion of them is attended with a threatening of suffocation.* How can a professor be ignorant of such things, when every tyro fixes on his memory the sententious truths which the immortal Cullen taught, and which inform us, that the first feature of the phlegmasiæ is *pyrexia;* and that the prominent phenomenon of pyrexia is "*viribus,* præsertim artuum *imminutis.*" Even if the statement which Dr. Hamilton makes were as true as we know it to be the reverse, still, we ask, would it not be a *mere exception* to a general rule, although he most disingenuously endeavours to couple and contrast it with another general rule, respecting mercury, which, by the bye, is nearly as erroneous as that on which we are animadverting.

"From the time that the influence of mercury becomes evident, *the general strength declines rapidly.*" Can Dr. Hamilton have the hardihood to make this assertion in the face of the profession, thousands of whom have seen their patients, while with their gums actually sore, and consequently where the "influence of the mercury was evident," go through their usual occupations—nay, the toils of naval and military duty, for weeks or months, with scarcely any perceptible diminution of strength. Every body knows, indeed, that when a man, say for a chancre, is put upon a course of mercury, and confined, as he should be, to the house, with low diet, while the perspiration and other secretions are augmented, he will *gradually,* not rapidly, get thinner in flesh, and more irritable in temper. But would not any reasonable man be ashamed of torturing this circumstance into such an exaggerated misrepresentation, as the concluding paragraph of Dr. Hamilton's quotation exhibits.

All men are aware that in peculiar idiosyncrasies of constitution, and in certain highly nervous and irritable habits, the effects portrayed by Dr. H. as general occurrences, will occasionally take place. But what will the state of practice be, if we calculate on these consequences as likely *always* to happen, when we know that, on an average, they do not happen once in one hundred times, where common prudence guides

---

* "Hinc est ut pleuritide in pejus ruente, difficilior evadit respiratio et prope mortem constitutis difficillima." *Baglivi, Lib. I, Cap. IX.*

the administration of the medicine? Why, the answer is, and in fact this is what Dr. H. is evidently aiming at, that mercury must be discarded from the list of medical agents—excepting in a single disease!

Dr. H. proceeding in his career of converting exceptions into general rules, lays down the following position.

"If there be ulcerations in any part of the body, they must as certainly degenerate into malignant sores (under the influence of mercury) as blistered surfaces or scarifications mortify in cases where the living powers are much exhausted." 9.

Thus with one hypothetical dash of his pen, our author, in all the dignity of doctorial dogmatism, annihilates the practical facts and sober deductions of Abernethy and the host of his followers, who, by alterative courses of the blue pill, have removed myriads of "ulcerations" and other cutaneous defœdations, dependant on depraved states of the gastric, biliary, and intestinal secretions!

We will not follow our author through all his laboured researches after chaff, to the exclusion of the grain; a single specimen of the "frightful" effects of mercury, collected and carefully recorded by our author, will be quite sufficient to excite the risibility of the profession, and the terrors of all the old women in England, Scotland, and Berwick-upon-Tweed.

"A lady, the mother of four children, in the twenty-eighth year of her age, had a bad miscarriage at the end of the fourth month. When the author was called, she was very much reduced from the loss of blood, and required the ordinary palliative remedies. Three days after the first visit, she complained of a bad taste in her mouth, with soreness of her gums, and on the following day salivation took place. On inquiring into the circumstances of her previous history, it was learned that *four years* before, she had had, for a fortnight, a course of the blue pill, which had only slightly touched the gums, and it was solemnly asserted that she had never again taken any preparation of mercury, and had been in general good health."—"The salivation, with the usual consequences of excessive emaciation, debility, and irritability, *continued for above twelve months.* (! ! !) Occasionally, for a day or two it was checked; but alarming *vomiting*, with threatening sinking of the living powers, supervened." 27, 28.

Really we could scarcely preserve our gravity while reading the above passage, in which Dr. Hamilton evinces either a great lack of knowledge, or superabundance of prejudice. He appears to have no idea that ptyalism can arise from any other cause than the introduction of mercury into the system, although Cullen and other nosologists have enumerated nearly thirty species of idiopathic or symptomatic salivation, independently of mercury. We have at this moment, under our eye, a lady, who, since the cessation of the catamenia, now nearly five years, has had a constant ptyalism, with swelled and even ulcerated gums. This exuberant secretion appears to affect the whole mucous membrane, from the mouth to the rectum; since she rarely passes a day without having from eight to twelve liquid, watery, and frothy motions, without any other bad effect than that of keeping her very weak, and disabling her from going into company, or even any distance in a carriage. The function of the kidneys is considerably reduced, and that of the skin almost entirely abolished; at least, no sensible perspiration is ever seen, and the cutaneous surface is rough and dry. When opiates or astringents are taken in such quantities as to check considerably the watery discharges from the bowels, then head-ache, vertigo, and other symptoms threatening apoplexy, supervene. This case affords a fine specimen of the vicarious or additional functions which Nature occasionally throws upon some organs or structures, when others are deranged or impeded. And, doubtless, Dr. Hamilton's patient, if examined with a philosophic instead of a prejudiced eye, would have exhibited some phenomenon illustrative of the principle in question, without leading the author into the monstrous error which the passage displays.

The second Section of Dr. Hamilton's work is on the powers of mercury in subduing the syphilitic poison, in which he appears most reluctantly to acknowledge that this pernicious mineral "has certain medicinal virtues," at least in one disease.

After some desultory reasonings our author comes to the following conclusions:

1st. That primary sores ought not to be dressed with mercurial applications, till their real nature be ascertained.

2d. That, every possible precaution should be taken, during the treatment of syphilis, to guard against the occurrence of general or local inflammation, by confinement within doors, quiet, warmth, vegetable food, &c.—a rule that every one knows well.

3d. That, with the above precautions, "a much milder course of mercury is sufficient for the cure of syphilis than has, in general, been hitherto supposed."

4th. That sarsaparilla, guaiacum, &c. are not superior in efficacy to water gruel.

While paying a just tribute of praise to the liberality of sentiment which pervades the circular instructions of Sir James M'Grigor to the army surgeons, on the important subject of treating syphilis without mercury, Dr. Hamilton "enters his most solemn protest against the introduction of this experiment into private practice."

"Patients (says he) in the ordinary ranks of society cannot possibly be kept under the same strict regulations and unremitting vigilance of superintendance with soldiers in a military hospital. It would be absurd to expect, that even the majority of persons in the higher stations would submit to confinement to bed, perfect quietude, and vegetable diet, for any length of time." 47.

In reply to Mr. Rose's supposition, that the affections of the bones, &c. which were so common, and so much dreaded, both by patients and practitioners, in former times, were the effects of mercury, and not of syphilis, our author observes:

"But all this is contradicted by the description of the ravages of that disease when first noticed, and before the use of mercury was resorted to; and by the testimony of Dr. Fergusson and of Mr. Guthrie, that at present in Portugal (where no mercury is employed) there are numerous individuals of all ranks with mutilated noses, and other personal defects, from the disease in question; and also by the fact, already specified, that in India, where such long continued courses of mercury are prescribed for affections of the liver, no disease of the bones, excepting the teeth and alveolar processes, was even known to occur under such circumstances." 49.

As an argument against attempting to cure really syphilitic complaints without mercury, Dr. Hamilton brings forward some curious and interesting matter relative to the liability of the infant *in utero* to be infected with the syphilitic virus, and to be destroyed by that poison long before the ordinary period of birth. Monsieur Bertin, Physician to the Venereal Hospital in Paris, asserts, that between 1801 and 1810, one thousand four hundred and fifty infected infants had been treated there. Of these, one thousand and eighty-five had been born either in that hospital, or in the MATERNITE, and therefore had been infected by their parents; and three hundred and sixty-five were brought from the city or country, many of whom had had the disease from nurses.

"Mons. Bertin has adduced numberless proofs that the infants in whom the disease appeared soon after birth, infected the nurses, and that these communicated the disease to their husbands." 52.

"That the male parent, although apparently in health, and while the female parent is totally free from disease, may infect the infant, Mr. B. Bell has taken particular pains to prove; and he has stated several cases in illustration, some of which fell also under the notice of the late Dr. Alexander Hamilton, and of the author of these pages. The concurrent observations of many other practitioners warrant the conclusion that, in married persons, the syphilitic virus may lurk for a considerable time in the habit of the male, without at all affecting his health, and without communicating any disease to the female, and may, nevertheless, infect the offspring of the parties." 56.

Dr. H. states that a late practitioner in Edinburgh acquired the reputation of curing syphilitic disorders very rapidly, by a liberal application of caustic to the primary sores, a few mercurial pills, and abstinence from intoxicating liquors. But the wives of the gentlemen so cured, repeatedly miscarried of dead infants. Although there can be no doubt that the mother, labouring under syphilis, may communicate it to the embryo; yet, our author thinks the poison generally lurks in the male parent. He considers that the disease is never cured in children thus infected, without mercury.

"The virus in this state (says Mr. B. Bell) is of a more deleterious nature than it ever appears to be in any other form of the disease. Besides being more particularly apt to communicate the infection, it proceeds with more rapidity to destroy the constitution, insomuch that if mercury be not immediately employed on the disease being perceived, it usually makes such quick progress, that a fatal termination can scarcely be afterwards prevented."

Dr. H. therefore considers it established by innumerable facts, "that syphilis, when a congenite affection in infants, cannot possibly be conquered by the natural powers of the constitution."

"Where the male parent alone had formerly had the complaint alluded to, it was the practice of the late Mr. Bell, and of Dr. A. Hamilton, to put both parties under a course of mercury; and the result certainly has been uniformly successful, the future progeny of the individuals having been born alive and healthy." 63.

Our author states that some cases occurred several years ago, in his own practice, where, from circumstances, it was impossible to adopt the above plan, and he was compelled to limit the use of mercury to the male parent. He had the satisfaction, however, of finding the experiment completely efficacious; and since then he has followed the same rule "wherever he had conclusive evidence that the female parent had never had any suspicious symptoms." In some instances, where the female had repeatedly borne dead children, although she herself had never had any syphilitic complaint, mercury was exhibited to her alone, during pregnancy; "but the effect of the practice has not been so invariably fortunate as in the former." Some of the infants were still-born; others, soon after birth shewed symptoms of the disease, which soon proved fatal; "and a certain proportion were born healthy, or had the complaint in so slight a degree, that it yielded to the appropriate remedies."

"In one case (where both parties had had the complaint, but were apparently cured) the mercurial course, by the prejudice of the parties, was not continued for the length of time recommended by the medical attendants. The lady, soon after, fell with child, and was delivered, between the seventh and eighth month, of an infant, who infected three healthy nurses in succession. The parties, in that case, eventually submitted to a proper mercurial course, and afterwards had three healthy children." 65.

While, then, death is almost certain, when children, affected with congenite syphilis, are not treated by mercury, so, "on the other hand, a large proportion is saved by that mineral." Mons. Bertin informs us, that 400 out of 1069 infants, treated in L'Hopital des Veneriens, were cured by means of mercury, exhibited directly to themselves, or mediately through the nurse.

"If the facts detailed in the preceding pages, respecting the liability of the infant in the womb to the infection of syphilis can be depended upon, and he ventures to assert that they can be neither controverted nor extenuated, the irresistible conclusion must be, that mercury affords the only safe and efficacious remedy for complaints really syphilitic." 67.

Such were our own sentiments at the commencement of this Journal; but we have seen such documents as induce us to suspend our opinion on the question at present, confident that the *experimentum crucis* is in the hands of men as able to decide with precision, as they are honourable in reporting with candour, the result of this momentous trial.

The third Section, on the cautions necessary during the use of mercury, contains a code of judicious rules, but none of them unknown to any well-informed practitioner. Confinement within doors; low and mild diet; blood-letting, if the patient be robust; gradual introduction of the mercury into the system, especially the quicksilver pill and mercurial ointment—not carrying the saturation beyond a brassy taste in the mouth;* diluent drinks, particularly the decoctum sarsæ; *daily* use of the warm bath, where convenient;† suspension of the medicine, in case of any feelings of morbid irritation; duration of the course for two or three months, according to circumstances; and confinement to the house

* We conceive this degree of mercurialization somewhat too low, especially in secondary and obstinate affections. Rev.

† This, also, we believe to be objectionable. It hurries off the mercury too quickly through the pores of the skin. Rev.

for a fortnight after the medicine is left off, with great attention to warm clothing subsequently, form the whole pith of this section.

The fourth Section is on the means of relieving the morbid effects of mercury; and contains little that is satisfactory. It is well known that John Hunter depended chiefly on country air and cold bathing. Messieurs Abernethy, Mathias, and Pearson, recommend sarsaparilla, cicuta, nitric acid, country air, and generous diet; Mr. Carmichael, preparations of antimony. Much may be done, as Dr. Hamilton properly observes, by the regulation of the diet, and the mental and corporeal exercise, with the occasional exhibition of vegetable tonics.

"In very young subjects, dry friction, and the internal use of preparations of iron, seem to have the happiest effects."

Section 5. *On the Use of Mercury in Liver Diseases.* Dr. Hamilton observes, that experience has established the efficacy of mercury in arresting the progress of inflammation of the liver in hot climates. He errs, when he says that the *violence and rapidity* of the symptoms, in tropical climates, have led to the most active modes of exhibiting mercury there. His remarks on organic diseases of the liver are so exceedingly vague, erroneous, and conjectural, that we will not waste time upon them, especially as we know that ere these sheets see the light, a work will appear, containing observations on these diseases, of a very different character from Dr. Hamilton's lucubrations.

In the sixth Section, "*on the Use of Mercury in Affections of the Stomach,*" Dr. Hamilton fights manfully against a shadow, and is triumphant. No man, in his senses, would think of salivating a patient for pure dyspepsia; but as, in many cases, this same dyspepsia is symptomatic of irregular or defective secretion in the liver, we maintain that alterative doses of the quicksilver pill, combined with gentle eccoprotic medicines, are exceedingly serviceable; and we only wish we had an opportunity of measuring the success of this practice with that of Dr. Hamilton.

In page 103 of this Section, we have a curious specimen of Dr. Hamilton's pharmaceutical knowledge.

"There is good cause for the popular prejudice against *aloetic* medicines, that they are apt to produce hæmorrhoids. *Such* medicines, therefore, are only to be employed pro re nata; and for an *ordinary* laxative, the *compound extract of colocynth* is much preferable."

Every reader knows that *one half* of the solid ingredients in this ordinary substitute for *aloetic* medicines is ALOES! Such is the accuracy of a man, who modestly tells us a few pages before, that "it is the object of the author to improve the *science of medicine.*" The extract above mentioned presents the only novelty which we met with in this section of the work.

In the seventh Section, Dr. Hamilton endeavours to draw the attention of the Faculty to "an affection of the duodenum which imitates chronic hepatitis;" "and which, though very particularly described by Sylvius, Hoffman, and others, has been lately not much attended to." We much fear, that all Dr. Hamilton adds to our stock of knowledge on this subject, will have little effect in exciting to a renewed investigation; nevertheless, we are always eager to listen to any thing in the shape of practical matter.

The disease under consideration, Dr. H. thinks, consists at first in a diminished power of the duodenum; in consequence of which the food, in passing through it, does not undergo the usual healthy changes. After a certain period, however, the organ becomes so tender, that pain is occasioned both by distension from within, and compression from without. It is in this stage, according to our author, that the disease may be confounded with hepatitis, "the symptoms of the two diseases so nearly resembling each other, that it is scarcely possible to discover the diagnostic marks peculiar to each." Dr. Ferriar, in his Medical Histories, &c. has given us the following diagnostics on this point. When the duodenum is affected—

"The pain may be felt to change its place a little on the expulsion of wind. The pulse likewise is soft, though very irregular. The secretion of mucus from the Schneiderian membrane is interrupted, and sometimes nearly ceases. He is generally, but not obstinately costive, and subject to torpor and nervous oppression. A slight inflammation of the fauces also attends the disorder, returning once in eight or ten days." *Vol. ii.*

The same author remarked, too, that before the exacerbation of pain in this disease, the urine is of a bright green colour. Dr. H. does not place much confidence in Dr. Ferriar's diagnostics, nor does he agree with Mr. Abernethy, in considering the appearance of the stools as affording the best evidence of the true condition of the liver. He even believes "that several of the cases detailed by Mr. Abernethy, as instances of affections of the liver, were, in fact, disorders of the duodenum." Dr. H. like many others, perpetually confounds functional with organic disease in the liver. Thus, granting that an irritable or sub-inflamed state of the mucous membrane of the duodenum were present, is it not highly probable, that, either as cause or effect, a depraved state of the biliary secretion accompanies it? But as long as the liver cannot be felt like a large cheese, in the right hypochondrium, there can be no "liver disorder," according to the confined notions of some physicians. We have, in former numbers of this Journal, stated our conviction that the colour and appearance of the stools are greatly affected by morbid secretions from the internal surface of the intestines themselves; and therefore we are very far from attributing every deviation from a natural state in them to a morbid secretion of bile. Nevertheless, we are firmly persuaded, from no common attention to the subject, that the fæcal excretions are, upon the whole, the main index of the state of the biliary secretion; and consequently we consider Dr. Hamilton's depreciation of Mr. Abernethy's observations, as a branch of that system of prejudice which manifestly pervades the whole work. We shall allow the author, however, to delineate his duodenology in his own words.

"On this principle, in doubtful cases, where, along with lassitude, sallowness of the complexion, occasional sickness, total want of appetite, thirst, irregular state of the bowels, occasional palpitation of the heart, flatulency, frequency or irregularity of pulse, dull pain in the right side, extending to the top of the shoulder, aggravated by pressure, by exercise, or by lying on the left side, and becoming occasionally acute after taking food, with loss of flesh, if there be a daily evacuation from the bowels exceeding considerably in the quantity of real fæces the food actually swallowed, the author considers the disease to depend on the state of the duodenum, if there be no tangible hardness or enlargement of the liver. When, along with the same symptoms, there is a milky white appearance of the urine, as if it were mixed with chalk, he never has any doubt on the subject, because he has invariably found the duodenum affected under such circumstances, and he never observed the same appearance of the urine in diseased liver. He owns, that in several cases where the duodenum was certainly affected, that peculiar state of the urine did not attend." P. 110.

The fæces, Dr. H. states, have been reported to be of a chalky colour, by some practitioners, in consequence of having been covered with the urine, and it was necessary to separate the two excretions before the error was acknowledged. The bright green colour of the urine, mentioned by Dr. Ferriar as preceding the exacerbation of pain in affections of the duodenum, has not come under the author's notice.

In some cases, Dr. H. observes, the pain in the duodenum is characterized by its occurring commonly about an hour after eating—by its being different from that arising from distention of the stomach from flatulence, and by its not being relieved by the expulsion of wind by eructations.

"Individuals who have resided long in warm climates, and have suffered from affections of the liver, are particularly liable to this disorder of the duodenum." P. 113.

This affords very presumptive evidence, that in all climates the complaint is rather a consequence of disordered hepatic secretions poured into the duodenum, than a cause of the liver affections.

If Dr. Hamilton has laboured hard to explode mercury from general use, he has endeavoured to make amends for this by recommending another article from the mineral kingdom, which, with all the virtues of mercury, is devoid of any of its pernicious qualities. Our readers will be exceedingly impatient to know what this is; and we shall not keep them in suspence—it is—MURIATE OF LIME!

"Nature has furnished a most efficacious remedy for the removal of that *tendency* to diseases of the liver, which is so apt to occur in Europeans who have inhabited hot climates—viz. the muriate of lime, which forms the active ingredient of almost all the natural mineral waters." P. 114.

Our author gives us the following agreeable intelligence.

"If, instead of the Cheltenham water, the muriate of lime be taken in doses adapted to the constitution of the patient, and be steadily persevered in, *there can be no risk of disappointment.*"

It is a little unfortunate, however, that, "as this medicine produces no sensible operation," "the impatience and the prejudice which so commonly attend a broken state of health, make invalids apt to become tired of it." This we can very easily believe, and as the subject is beneath notice, we shall waste no further space on the muriate of lime.

It will be remembered by our readers that in a late number of this Journal, we noticed the scepticism of Dr. Bateman relative to the operation of the nitro-muriatic bath applied to the surface of the body; that physician maintaining, that it was *chemically* and *physiologically* impossible that the said bath could have any effect, bad or good. We cite the two following passages for the perusal of Dr. Bateman and those chemico-physiological sceptics who, in their closets, decide on the efficacy or inefficacy of a medicine, by means of chemical and physiological theories, in opposition to clinical observations and facts.

"The nitro-muriatic-acid bath exerts a powerful stimulus on every part of the alimentary canal, and when prudently employed, is most useful in particular complications of disorder; but wherever there is a tendency to inflammation in any part of the chylopoietic viscera, it cannot fail to aggravate the symptoms." 116.

The next passage forms a pretty significant commentary on the *inertness* of this remedy.

"Professor Burns, of Glasgow, (on whose veracity and practical acumen it is unnecessary to expatiate), mentioned some time ago in a private communication to the author, that the use of the nitro-muriatic-acid bath has a tendency sometimes to excite a discharge of blood from the coats of the stomach, inducing vomiting of a fluid something like hare-soup, mixed with dark coagula, which the patient and attendants suppose to be vitiated bile, and do not consider to be dangerous, although death invariably follows in a short time. In the instances of this kind which have fallen under Mr. Burns's notice, the acid bath had not been employed with his sanction; and from what has been already stated, there is every probability that the original disease, in most of those cases, had been the affection of the duodenum alluded to, and not a disease of the liver." 116, 117.

For the cure of this disorder of the duodenum Dr. H. observes, that different means are required, according to its progress.

"In slight cases, repeated doses of rhubarb, combined with magnesia or other laxatives, and a course of the white oxyde of bismuth, with mild food, are generally sufficient. But in the more violent degrees, it is necessary to begin with the application of leeches to the epigastric region; after which one or more antimonial emetics must be directed, and nauseating doses of the same, with occasional laxatives, ought to be continued for some time—the patient being kept all the while on the lowest possible diet, with respect both to the quality and the quantity of the food. After the urgent symptoms have yielded, the same mode of diet as that recommended for the cure of dyspeptic complaints ought to be adopted, and the white oxyde of bismuth, combined with some aromatic, is to be advised." 118.

In the eighth Section of the work we only find a conjecture, that "in those affections of the spleen or pancreas which follow remittent or intermittent fevers, it seems *probable* that a continued course of super-tartrite of potass, or of the *muriate of lime*, combined with antimonials, with a proper regulation of the diet, would be both a safe and a beneficial mode of practice."

The ninth Section, on the use of mercury in affections of the intestinal canal, is, as usual, overflowing with prejudice. Because nurses and rash practitioners will administer calomel in profusion to children, and thereby do mischief, are we to blind our mental optics to the beneficial effects of the same remedy, when prudently administered? Even the tartrite of antimony, which Dr. Hamilton would wish to substitute in all cases for calomel, is not devoid of danger, when incautiously given to children, as was long ago remarked by the late Dr. John Clarke.

The following extract will shew how Dr. Hamilton's universal CATHOLICON, the muriate of lime, is to be administered.

"Half a drachm of the ordinary solution of that salt, mixed with eight or ten ounces of water, agreeably sweetened, may be taken twice a day, for a great length of time, without producing any immediately sensible operation; and in most instances, with an obvious improvement of the general health. But if three or four drachms of the same solution be given as a dose, a most violent debilitating diarrhœa is commonly excited; and if the same dose be persevered in, for even a few days, the most alarming emaciation speedily follows." 136.

Dr. Clarke, of Dublin, among innumerable others, having *experienced* the good effects of small doses of calomel, "both in the bilious diarrhœa of adults, and in the green scour of young children," Dr. Hamilton sets about disproving these *facts* by a chain of his usual rhetoric.

"But it has always appeared to the author, that wherever looseness is accompanied with an increased flow, or any unusual acrimony of the bile or of the intestinal secretions, evinced by the appearance of the stools, which are bilious, or acrid and watery, or slimy or bloody, or dark coloured and offensive, preparations of mercury, however small the dose may be, add greatly to the irritation, unless they happen to pass unchanged through the alimentary canal. As he has been called in to several cases where young children had most obstinate and violent diarrhœa in consequence of mercurial friction, and as he has repeatedly seen ulcerations on the surface of the colon, where small doses of calomel had been employed for a considerable time in order to moderate habitual diarrhœa in infants, he has been led to suspect, that upon many subjects, the calomel, from being involved in the contents of the stomach and bowels, or from some other cause, does *not act at all as a medicine.*" 138.

The idea conveyed in the last line is too good not to be improved upon; accordingly our sagacious author concludes at once, that in all cases, where calomel is taken with impunity, or *appears* to do good, it is by passing through the bowels unchanged, and doing nothing at all!

"Should this be admitted, a ready explanation is afforded of the impunity with which, in many instances, calomel is administered; and "*on the same hypothesis*, it may be understood, that small doses of calomel may *seem* useful in bowel complaints, when in reality the amendment is to be attributed to the regulation of the diet, and to the opiates which are commonly recommended at the same time." 139.

Our author having thus ascended to the climax of error and prejudice, we shall leave him to descend by his own weight, as he certainly requires no other impulse.

The deserts of Africa have their Oases—the darkest night has its gleams of star light—the most abandoned libertine feels, occasionally, remorse of conscience—and Dr. Hamilton's "abuse of mercurial medicines," contains some truths.

"There is, however, one variety of diarrhœa, common both to adults and to children, in which preparations of mercury appear to give relief more speedily and more certainly than any other medicines. It usually supervenes to cases of protracted indisposition, and is characterized by the fæces being very liquid, and of a light clay colour with a most offensive smell, and by the extreme debility which attends. This variety of diarrhœa seems to be owing partly to some irregular action of the liver, and partly to some unusual state of the great guts. That the functions of the liver are interrupted, is presumed by the diminished quantity of bile in the stools, and that there is some unusual state of the great guts, is proved by there being no solid fæces discharged. It may be, that the duodenum and other small intestines are also disordered, for it is not easy to explain the emaciation and the debility on any other supposition.

"Probably the alteration in the state of the great guts, is rather in the secretion from their internal surface, than in any change of texture, not only because the disease very often yields to medicines, but also, because in fatal cases, the whole track of those intestines has been found in a healthy condition, in respect to structure, although quite empty of any thing like solid fæces. From his reluctance to employ mercurial preparations, the author at one time tried a number of different remedies for the cure of this disorder; but he is from attentive observation convinced, that small doses of some of the mildest preparations of mercury, such as the blue pill, with or without ipecacuan, are singularly efficacious in such cases. Those medicines are to be repeated every eight or ten hours, or oftener, till healthy stools appear, and are then to be slowly withdrawn. If calomel be preferred to the blue pill, the dose ought never to exceed one grain, even to an adult." 140, 1.

Dr. Hamilton considers calomel as an efficacious, but, of course, a dangerous anthelmintic. He, therefore, proposes sea-salt, the muriate of lime, garlic, the semen santonicum, chamomile flowers, combinations of rhubarb, scammony, senna, gamboge; and more especially the rectified oil of turpentine mixed with cold drawn castor oil, as preferable to mercurial medicines. To this proposition we have no objection. Ascarides are easily dislodged by aloetic glysters, followed up, in ten or twelve hours, by a dose of jalap and senna.

In Dr. Hamilton's Section on Dropsy, there is very little new; and that little we suspect to be—not true. We may just mention, that the basis of his dropsical pathology is founded on Dr. Blackall's test of coagulable or non-coagulable urine; a test which is now proved by the most ample experience, to be totally fallacious, and inapplicable to practice.* His hypothesis then, being planted in error, can never rise up in truth: As an instance of the tissue of error and prejudice, which our author has industriously woven in this work, he asserts, that mercury is not only of no use in hydrocephalus, but that it often occasions the disease!

Speaking of ovarian dropsy, we find an interesting remark or two which we shall gladly quote.

"Adverting to the effects of percussion and of pressure in chronic rheumatism, and knowing the influence of the continued use of the muriate of lime in indolent glandular swellings, he was led to the trial of those several means, as being, at any rate, perfectly safe. He advised, therefore, that moderate and equable pressure of the abdomen should be made by means of a suitable bandage, that the enlarged part should be subjected twice a-day to gentle percussion, and that a course of small doses of the muriate of lime should be continued for at least several months. Where pain or tenderness was experienced on the ovary being pressed upon, he recommended, in addition to the above means, the daily use of the warm bath. This plan of treatment has been much more successful than he had anticipated. In seven cases, in which it has been tried, the enlargement has so completely subsided, that it is no longer tangible." 210.

The most interesting section in the work, is the 9th and last. On it, therefore, we shall dwell more particularly. It is on the use of mercury in croup and iritis.

About sixteen years ago, in consequence of the urgent representations of the late Dr. J. Anderson, but chiefly because the ordinary remedies frequently proved uncertain, our author was induced to give calomel a fair trial in croup.

"He can solemnly assert that, according to all that he has seen, no relief whatever has been afforded by that medicine, unless copious dark green coloured stools, like boiled spinach, have been discharged, and that it requires large and repeated doses of the medicine to produce that effect; for example, to a child of seven years old, one hundred and thirty-three grains were given within sixty hours. These circumstances lead to the conclusion, that in this part of the world, at least, wherever calomel has seemed to cure croup without affecting the bowels, the symptoms had not been those of that disease, but of the spurious croup; and this opinion is confirmed by the fact, that in the only cases in which the medicine has failed under the author's direction (being in the proportion of

* See our Fifth Number, Review of Dr. Crampton on Dropsy.

four out of fifty) no evacuation whatever through the bowels could be produced, although antimonials, and jalap, and gamboge, and glysters were employed as auxiliaries." 206.

The foregoing quotation, shewing that calomel saved after the rate of forty-six in fifty, offers, one would suppose, a favourable view of this *poison*. Dr. H. is utterly astonished at—"the safety with which a hundred and thirty grains of calomel could be given in this climate, within sixty hours, to a patient of seven years of age"; and well he may, if one fiftieth part of his abuse of the medicine were fact. Speaking of "the relief which has invariably followed the discharge of the dark-coloured evacuation," our author observes—

"This latter circumstance, in particular, is the more perplexing, seeing that, in all those cases, the evacuations from the bowels were not fæces, but were combinations of bile and mucus in a substantial form, resembling, as already stated, boiled spinach, or the contents of the second stomach in ruminating animals."* 208.

Dr. Hamilton thinks there is no reason to suppose so direct a sympathy of the larynx with the liver, as the above observations would induce one to suspect; for—"the immediate operation of calomel, where successful in croup, is most obviously on the liver." We may refer our readers to page 352 of our first volume, (Quarterly Series) for the sentiments of Dr. Wilson on this subject. That ingenious physician "is convinced, from a careful attention to the phenomena of croup, that the primary seat of irritation is in the digestive organs, whence is generated, by morbid sympathy, a spasmodic affection of the glottis, ending in inflammation of the mucous membrane of the larynx and trachea, ultimately destroying the action of the lungs by rendering them impervious. Acting from these premises, Dr. Wilson asserts that our great indication is to clear the stomach and bowels as early as possible, by emetics and mercurial cathartics."† Thus we see, that Dr. Hamilton's observations elucidate and confirm those of Dr. Wilson, though the former appears to know nothing of the writings of the latter.

To check the inflammation when once commenced, the lancet, however, must be used; and Dr. H. recommends the external jugular vein to be opened, in preference to the ap-

* This passage may reconcile Dr. Porter's observations respecting the power of calomel in changing the colour of the stools. We always thought and said, that it *dislodged*, rather than produced these coloured matters. Rev.

† Medico-Chirurgical Journal, vol. i. p. 352.

plication of leeches, which may be used as auxiliaries. Our author judiciously advises the blisters to be applied to the sternum, rather than to the throat. Notwithstanding what Dr. Hamilton has stated above, yet such is the inveteracy of his prejudice, that he advises others *not* to follow the successful practice which he himself pursued—saving 46 out of 50—but to trust to bleeding, blistering, purging, and antimony—*without any calomel!*

Dr. H's observations on Iritis are on a par with the rest of the work. He labours to prove that Dr. Farre, Mr. Saunders, Mr. Guthrie, Mr. Travers, and various others, were all deceived in respect to the efficacy of mercury in inflammation of the iris; and "he is inclined to attribute the success chiefly to the external application of the extract of belladonna"!

It has been justly observed, that

"Quem deus vult perdere prius dementat."

Dr. Hamilton, in his furious and persecuting zeal against mercury, has been thrown so much off his guard—has committed such glaring blunders—uttered such palpable absurdities—and betrayed such blinded prejudice, that it was only necessary to allow him to flounce about till he became entangled in his own toils, and sunk exhausted under the weight of his own weapons.

Whenever he finds himself in a condition to renew the attack on a valuable medicine, and on what we consider to be sound principles of pathology, we shall be found at our post, and ready to do our duty.

